# A Rare Case of Adenomyoepithelioma of the Breast Complicated with Cyst Diagnosed by Preoperative Core Needle Biopsy: A Case Report

**DOI:** 10.70352/scrj.cr.25-0749

**Published:** 2026-04-24

**Authors:** Keiichi Takahashi

**Affiliations:** Takahashi Breast and Gastroenterology Clinic, Osaka, Osaka, Japan

**Keywords:** adenomyoepithelioma, fine-needle aspiration, core needle biopsy

## Abstract

**INTRODUCTION:**

Adenomyoepithelioma (AME) of the breast is a rare benign disease in which glandular epithelial cells and myoepithelial cells proliferate to form a mass. Preoperative diagnosis is considered difficult, and diagnosis by core needle biopsy (CNB) is particularly difficult. Due to its heterogeneous features and growth patterns of the tumor cells constructing the tumor mass, this tumor, although benign in nature, is misdiagnosed as ordinary breast cancer and often treated by surgery for breast cancer. Here, a rare case of AME of the breast complicated with cysts diagnosed by preoperative CNB is reported.

**CASE PRESENTATION:**

A 27-year-old female presented with a chief complaint of a lump in the upper-inner quadrant of the left breast that had been present for 6 months. Bloody discharge had been noted from the left nipple for 1 week prior to her visit. A mass was palpable on physical examination. Mammography and ultrasonography detected a cystic mass and a solid mass in the upper-inner quadrant of the left breast. Fine-needle aspiration of the cystic mass revealed blood-derived cells and foam cells. CNB of the solid mass in the left breast revealed small and large ducts within a cyst, with myoepithelial cells proliferating between them. The stroma exhibited mucinous edema-like changes. Immunohistochemical staining for p63 and alpha-smooth muscle actin confirmed the proliferation of myoepithelial cells. The diagnosis of CNB was benign AME. Subsequently, the left breast mass was resected. The final pathological diagnosis was benign AME.

**CONCLUSIONS:**

Here, a rare case of AME of the breast complicated with cysts diagnosed by preoperative CNB is reported.

## Abbreviations

α-SMA
: alpha-smooth muscle actin
ADC
: apparent diffusion coefficient
AME
: adenomyoepithelioma
CK7
: cytokeratin 7
CNB
: core needle biopsy
EMA
: epithelial membrane antigen
FNA
: fine-needle aspiration
HE
: hematoxylin and eosin
MMG
: mammography
SLNB
: sentinel lymph node biopsy
US
: ultrasonography

## INTRODUCTION

AME of the breast is a rare benign disease characterized by the proliferation of glandular epithelial cells and myoepithelial cells to form a mass.^1,2)^ Preoperative diagnosis is considered to be difficult, and diagnosis by CNB is particularly difficult.^2,3)^

AME of the breast is classified as a benign tumor. However, cases of malignant transformation have been reported, although they are rare. In such cases, the prognosis is often poor.^4)^ According to previous reports, the percentage of patients with benign AME of the breast exhibiting malignant findings is 38%–50% by palpation, 60%–81% by US, and 44%–62% by MMG.^5,6)^ The correct diagnosis of benign AME is difficult in FNA as well.^7)^ On cytological assessment, 30%–45% of benign AME cases are classified as class IV or V. Therefore, histological confirmation using immunohistochemistry for myoepithelial markers is essential for a definitive diagnosis.^5,6)^ This tumor is misdiagnosed as ordinary breast cancer and therefore often treated by surgery for breast cancer.^1,3)^ When diagnosed with AME, complete surgical resection is important, and careful follow-up is required. No established drug therapy is available.

## CASE PRESENTATION

**Fig. 1:**
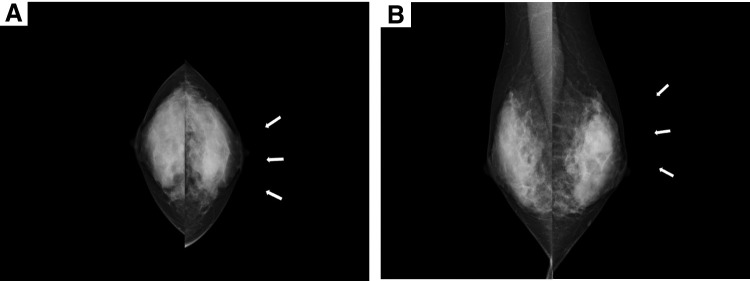
MMG (**A**, **B**). MMG revealed a lobulated dense mass with a partly ill-defined border in the left MU-I region as indicated by the arrows, which was a category 3 finding. MMG, mammography

**Fig. 2:**
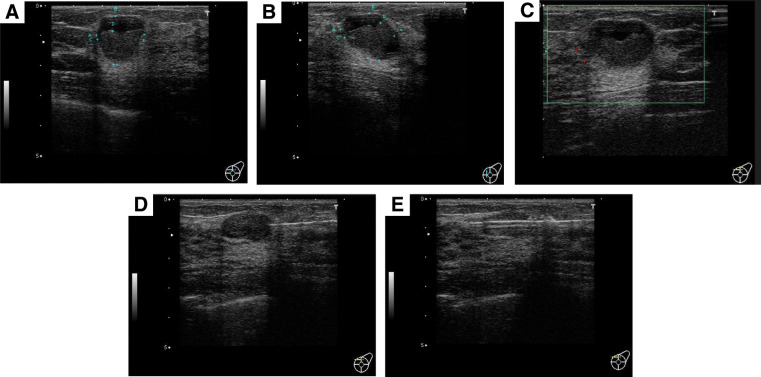
Breast US (**A**–**E**). US revealed a cystic mass measuring 16.8 × 21.4 × 16.1 mm and a solid mass measuring 16.3 × 17.4 × 12.2 mm in the upper-inner quadrant of the left breast. Color Doppler US showed a peripheral vascular enlargement and a lack of central vascularization. The core needle was inserted parallel to the chest wall and was fired as the needle tip reached the edge of the tumor so as to keep the tumor from shifting. US, ultrasonography

A 27-year-old female with no remarkable medical or family history presented with a chief complaint of a lump in the upper-inner quadrant of the left breast that had been present for 6 months. Bloody discharge had been noted from the left nipple for 1 week prior to her visit. On physical examination, a mass was palpable in the upper-inner quadrant of the left breast. MMG revealed a lobulated dense mass with a partly ill-defined border in the left MU-I region, which was a category 3 finding (**Fig. 1A** and **1B**). US revealed a cystic mass measuring 16.8 × 21.4 × 16.1 mm and a solid mass measuring 16.3 × 17.4 × 12.2 mm in the upper-inner quadrant of the left breast. Color Doppler US showed a peripheral vascular enlargement and a lack of central vascularization (**Fig. 2A**–**2C**).

FNA of the cystic mass in the upper-inner quadrant of the left breast was performed carefully to avoid damaging the solid tumor tissue by ensuring the needle did not penetrate it, puncturing and aspirating only the cystic fluid portion to prevent adverse effects on subsequent CNB diagnosis.

FNA revealed blood-derived cells and foam cells. No epithelial component was found. A cystic lesion was suspected. Since a small number of degenerate cells with densely stained nuclei were also observed, continued follow-up observation was considered desirable. Benign cystic lesions were suspected. The CNB procedure was performed securely and accurately following basic techniques. The core needle was inserted parallel to the chest wall to apply equal pressure to the tumor through the skin and was aimed at minimizing tissue damage when the core needle penetrated through the tumor due to tumor dislocation. The core needle was fired as the needle tip reached the edge of the tumor so as to keep the tumor from shifting (**Fig. 2E** and **2F**).

CNB of the solid mass in the left breast revealed small and large ducts within a cyst, with myoepithelial cells proliferating between them. The stroma exhibited mucinous edema-like changes. Although malignancy was negative, immunostaining was performed, and the results were reported additionally.

**Fig. 3:**
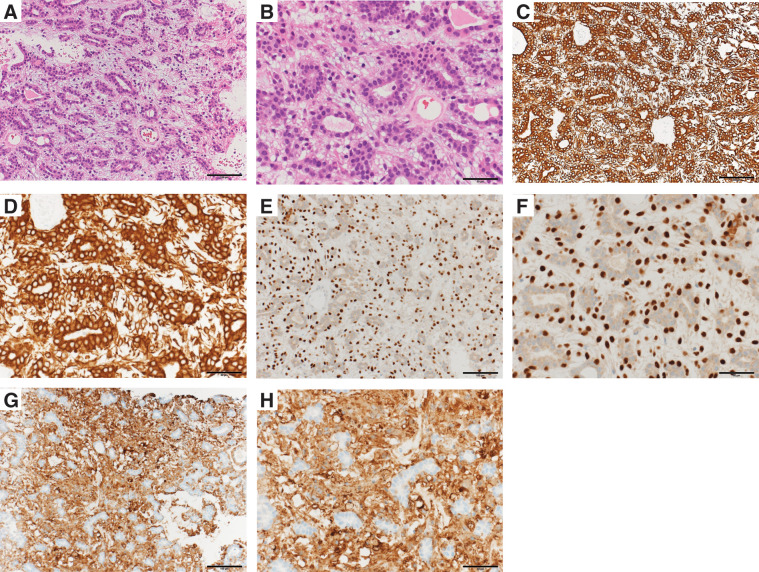
Pathological analysis. (**A**) HE stain, ×200, (**B**) HE stain, ×400, (**C**) CK7 stain, ×200, (**D**) CK7 stain, ×400, (**E**) p63 stain, ×200, (**F**) p63 stain, ×400, (**G**) α-SMA stain, ×200, (**H**) α-SMA stain, ×400. (**A**, **B**) HE staining revealed ductal epithelial cells with minimal atypia forming small tubules, surrounded by proliferating myoepithelial cells (HE ×200, ×400). (**C**, **D**) CK7 staining showed strong positivity in the cytoplasm of glandular epithelial cells and weak positivity in surrounding cells (CK7 ×200, ×400). (**E**, **F**) p63 staining showed that cells surrounding the lumen exhibited p63 positivity in their nuclei and were considered myoepithelial cells (p63 ×200, ×400). (**G**, **H**) α-SMA staining demonstrated that myoepithelial cells (α-SMA–positive) were predominantly proliferating, suggesting a tubular adenomyepithelioma (α-SMA ×200, ×400). α-SMA, alpha-smooth muscle actin; CK7, cytokeratin 7; HE, hematoxylin and eosin

HE staining revealed ductal epithelial cells with minimal atypia forming small tubules, surrounded by proliferating myoepithelial cells (HE ×200, ×400) (**Fig. 3A** and **3B**).

CK7 staining showed strong positivity in the cytoplasm of glandular epithelial cells and weak positivity in surrounding cells (CK7 ×200, ×400) (**Fig. 3C** and **3D**).

p63 staining showed that cells surrounding the lumen exhibited p63 positivity in their nuclei and were considered myoepithelial cells (p63 ×200, ×400) (**Fig. 3E** and **3F**).

α-SMA staining demonstrated that myoepithelial cells (α-SMA–positive) were predominantly proliferating, suggesting a tubular AME (α-SMA ×200, ×400) (**Fig. 3G** and **3H**).

Immunohistochemical staining for p63 and α-SMA confirmed proliferation of myoepithelial cells. It was considered to be AME.

**Fig. 4:**
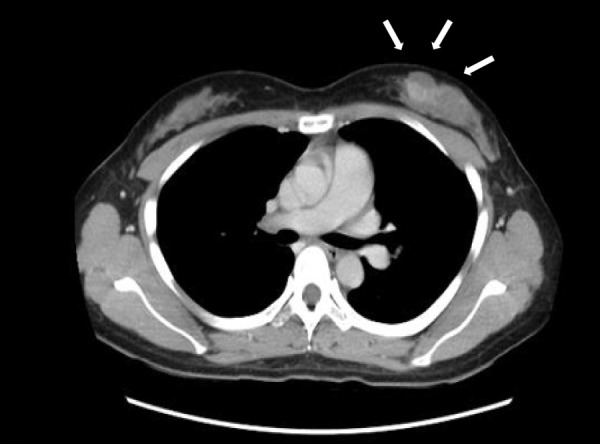
CT. CT examination showed a mass measuring 2 cm in diameter was present in the upper-inner quadrant of the left breast as indicated by the arrows. There were no findings suggestive of obvious lymph node enlargement or distant metastasis.

**Fig. 5:**
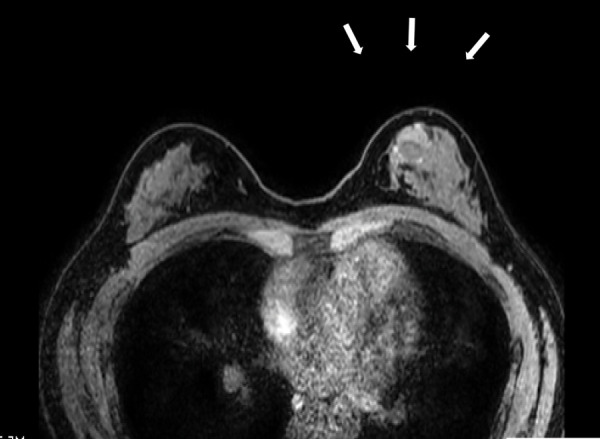
MRI. MRI examination showed a space-occupying lesion measuring approximately 2 cm in diameter in the upper-inner quadrant of the left breast as indicated by the arrows. There were no findings suggestive of obvious lymph node enlargement. The lesion was well defined, and a cystic mass was seen.

On CT, a mass measuring 2 cm in diameter was present in the upper-inner quadrant of the left breast. There were no findings suggestive of obvious lymph node enlargement or distant metastasis (**Fig. 4**). MRI revealed a space-occupying lesion measuring approximately 2 cm in diameter in the upper-inner quadrant of the left breast (**Fig. 5**). There were no findings suggestive of obvious lymph node enlargement. The lesion was well defined, and a cystic mass was seen. It appeared to be composed of a ventral cystic part and a solid dorsal part. The cystic area was associated with heterogeneous high signal intensity on T1-weighted images and a high-to-low signal intensity gradient or fluid surface formation on T2-weighted images, reflecting the existence of a hematic cyst. The solid part showed restricted diffusion, early staining, and equilibrium-phase washout (so-called rapid washout pattern). Based on the findings of the contrast dynamic study, AMEs were likely to be retained for the differential diagnosis of breast cancer. However, there was an impression that the possibility of malignancy could not be ruled out due to the presence of bloody nipple discharge (this seems unlikely to be reported in benign AME so far as examined).

The left breast mass was subsequently resected. The resection margin was 1 cm. The final pathological diagnosis after surgery was benign AME. The surgical margin was negative.

## DISCUSSION

AME is a rare tumor, accounting for 0.5% of breast tumors in peri- and postmenopausal women. Furthermore, cystic or prominent cystic changes are extremely rare.

Breast AME is a unique tumor that was first described by Hamperl in 1970.^8)^ While it most commonly arises in the skin, it can also occur in the mammary and salivary glands.^9)^

AME of the breast is a rare disease in which glandular epithelial cells and myoepithelial cells proliferate to form a mass. The age of patients ranges from 16 to 93 years, that is, from young adults to the elderly,^10–14)^ but the most susceptible age is considered to be 50 years or older. Also, male cases were reported.^15)^ The tumor size is between 0.3 and 8 cm, with a firm consistency, grayish-white color, and an average size of 2.5 cm.^16,17)^ It may sometimes show hemorrhagic permeation or cystic changes.^18)^

Most AMEs form solid masses rather than cystic masses^19)^; cystic or prominent cystic changes are extremely rare, but a few may contain small cystic areas.^20)^ A review of the literature has reported only 1 case of cystic AME.^21)^

Despite being mostly benign tumors, the rare case of AME of the breast has no specific imaging features. Therefore, preoperative diagnosis is difficult. The radiological findings of breast AME are nonspecific, and MMG is usually characterized by a noncalcified ovoid or lobulated mass with smooth margins.^18,22)^

Furthermore, MMG has been reported to show various forms of masses with well-defined to irregular margins and round to irregular shapes, and cases with calcification have also been reported. In breast US, lesions with varying morphological features are observed: well-defined and lobulated, intracystic tumors, and irregular-shaped lesions with heterogeneous internal structure.

On US, AME typically presents as a solid, hypoechoic, oval mass, often accompanied by posterior acoustic enhancement. Peripheral vascular enlargement is a feature, and Doppler US shows increased vascularization, mainly peripheral rather than central, or a lack of vascularization.^23,24)^

Due to its rarity and unusual presentation, it can mimic breast cancer on both the clinical and radiological levels.^25)^

MRI findings are variable, making differentiation from breast cancer difficult. Additionally, MRI detects masses that are round or irregular in shape, with smooth or irregular margins, heterogeneous enhancement, and delayed washout.^1)^ However, high ADC values on MRI and high signal intensity on T2-weighted images may be characteristic of AME. In some cases, tumors may be extensive and discretely distributed, making it important to evaluate the extent of lesions using MRI.

Although these lesions are benign and have a good prognosis, according to reports thus far, the percentage of patients with malignant findings has been reported as 38%–50% by palpation, 60%–81% by US, and 44%–62% by MMG.^4,5)^

The correct diagnosis of benign AME is difficult in FNA as well.^6)^ On cytological assessment, 30%–45% of benign AME cases are classified as class IV or V. Therefore, histological confirmation using immunohistochemistry for myoepithelial markers is essential for a definitive diagnosis.^5,6)^ In particular, diagnosis by CNB is difficult. One reason is that the tumor itself has heterogeneity, and tumor cells with varying growth patterns (e.g., lobulated, papillary, and tubular structures) are combined to construct a tumor mass.^2,3,26)^ Another reason is that the tumor cells readily form cell clusters.^13)^ Consequently, this disease is misdiagnosed as ordinary breast cancer and often treated by surgery for breast cancer.

The myoepithelial cells of AME of the breast are more abundant, thicker, and larger than those of other benign nodular adenopathies and intraductal papillary carcinomas.^18)^

Immunohistochemical staining for myoepithelial markers, especially p63, is useful for highlighting the abundant myoepithelial components.^18)^

Immunohistochemical staining was performed using α-SMA, S-100, EMA, CEA, cytokeratin, vimentin, and desmin; the results showed that α-SMA and S-100 had exclusive positivity in myoepithelial cells in all cases, while EMA showed exclusive positivity in glandular epithelial cells. For this reason, α-SMA, S-100, and EMA are considered useful for the diagnosis of AME.

Regarding pathological findings indicating malignancy of breast AME, Tavassoli reported that mitotic activity exceeding 10 mitotic figures (MF)/10 high-power fields (HPF) indicated severe cellular atypia suggesting malignancy.^16)^ Loose et al. highlighted the following 3 points: (1) mitotic activity of 11 MF/10 HPF or higher; (2) cellular atypia; and (3) invasive proliferation.^27)^ In past reports, the percentage of AME diagnosed as malignant or complicated with carcinoma was 25%.^18)^ In this case, benign AME was diagnosed due to the lack of histopathological findings suggestive of malignancy.

AMEs are classified as tubular, lobulated, or spindle cell variants on the basis of their growth patterns.^16,18)^

AMEs are generally benign neoplasms, although a small number of malignant lesions have been reported in the literature; either the epithelial or myoepithelial component may undergo malignant transformation.^28)^

Malignant transformation may occur in tumors larger than 1.6 cm.^10,29)^

Some papers have concluded that AMEs over 2 cm should be treated as malignant.^30)^

Malignant AMEs share morphological similarities with the related salivary gland tumors, which frequently harbor recurrent mutations in *HRAS* and *PIK3CA* genes.^31)^

Molecular studies reveal that malignant AME loses the ability to maintain normal cellular function due to frequent mutations in the phosphoinositide 3-kinase/AKT/mammalian target of rapamycin (PI3K/AKT/mTOR) signaling pathway (*PIK3CA*, *AKT1*, *HRAS*).^32)^

Recurrent hotspot mutations in *HRAS* Q61 and PI3K-AKT pathway genes function as oncogenic driver mutations directly involved in the initiation and progression of malignant AME.^11)^ This suggests that they are important targets for cancer therapy and potential therapeutic targets, necessitating further investigation and research.

Chromosomes 8 and 16 are both implicated in breast cancer, particularly mammary gland adenocarcinoma. In more detail, the balanced translocation between chromosome 8 and chromosome 16 alters the structure of the chromosomes, can change the arrangement of genetic information, and may affect gene expression. It has also been reported in benign AME.^33)^

For surgical treatment, resection with an adequate margin or partial mastectomy should be performed. An adequate margin is required because local recurrence is likely to occur even after surgical resection.^26)^ The risk of recurrence is high: 10% in benign and 35% in malignant cases.^4,34)^ Prognosis is worse if the myoepithelial component is predominant, especially in patients with high grade. Distant metastasis occurs most frequently in the lung (55%), followed by the brain (22%) and bone (11%).^35)^

If benign AME of the breast is diagnosed preoperatively, only lumpectomy with an adequate margin—that is, partial mastectomy—may be performed. Mastectomy, SLNB, axillary lymph node dissection, and radiotherapy may be omitted. No adjuvant therapy is required.

## CONCLUSIONS

AME of the breast is classified as a benign tumor. However, cases of malignant transformation have been reported, although they are rare. In such cases, the prognosis is often poor. Preoperative diagnosis of this disease is considered difficult. In many cases, it is misdiagnosed preoperatively as breast cancer and treated by surgery for breast cancer. If diagnosed preoperatively, only partial mastectomy may be performed. Not only mastectomy and SLNB but also axillary lymph node dissection and radiotherapy may be omitted. No adjuvant therapy is required. Here, a rare case of benign AME of the breast complicated with cysts diagnosed by preoperative CNB is reported.
